# NMR metabolite quantification of a synthetic urine sample: an inter-laboratory comparison of processing workflows

**DOI:** 10.1007/s11306-023-02028-4

**Published:** 2023-07-07

**Authors:** Cécile Canlet, Catherine Deborde, Edern Cahoreau, Grégory Da Costa, Roselyne Gautier, Daniel Jacob, Cyril Jousse, Mélia Lacaze, Inès Le Mao, Estelle Martineau, Lindsay Peyriga, Tristan Richard, Virginie Silvestre, Mounir Traïkia, Annick Moing, Patrick Giraudeau

**Affiliations:** 1 Toxalim (Research Centre in Food Toxicology), Toulouse University, INRAE UMR 1331, ENVT, INP-Purpan, UPS, MetaToul-AXIOM Platform, National Infrastructure of Metabolomics and Fluxomics: MetaboHUB, INRAE, 31027 Toulouse, France; 2 INRAE, Univ. Bordeaux, Biologie du Fruit et Pathologie, UMR1332, Bordeaux Metabolome - MetaboHUB, Centre INRAE de Nouvelle-Aquitaine Bordeaux, 33140 Villenave d’Ornon, France; 3 TBI, Université de Toulouse, CNRS, INRAE, INSA, MetaboHUB - MetaToul, National Infrastructure of Metabolomics and Fluxomics, 31077 Toulouse, France; 4 Univ. Bordeaux, Bordeaux INP, INRAE, OENO, UMR 1366, ISVV, Bordeaux Metabolome - MetaboHUB, 33140 Villenave d’Ornon, France; 5 Université Clermont Auvergne, Clermont Auvergne INP, CNRS, Institut de Chimie de Clermont-Ferrand. Université Clermont Auvergne, INRAE, UNH, Plateforme d’Exploration du Métabolisme, MetaboHUB Clermont, 63000 Clermont-Ferrand, France; 6 Nantes Université, CNRS, CEISAM UMR 6230, 44000 Nantes, France; 7 CAPACITES SAS, 44200 Nantes, France

**Keywords:** Metabolomic profiling, Peak integration, Deconvolution, Quantitative NMR, Synthetic urine

## Abstract

**Introduction:**

Absolute quantification of individual metabolites in complex biological samples is crucial in targeted metabolomic profiling.

**Objectives:**

An inter-laboratory test was performed to evaluate the impact of the NMR software, peak-area determination method (integration vs. deconvolution) and operator on quantification trueness and precision.

**Methods:**

A synthetic urine containing 32 compounds was prepared. One site prepared the urine and calibration samples, and performed NMR acquisition. NMR spectra were acquired with two pulse sequences including water suppression used in routine analyses. The pre-processed spectra were sent to the other sites where each operator quantified the metabolites using internal referencing or external calibration, and his/her favourite in-house, open-access or commercial NMR tool.

**Results:**

For 1D NMR measurements with solvent presaturation during the recovery delay (zgpr), 20 metabolites were successfully quantified by all processing strategies. Some metabolites could not be quantified by some methods. For internal referencing with TSP, only one half of the metabolites were quantified with a trueness below 5%. With peak integration and external calibration, about 90% of the metabolites were quantified with a trueness below 5%. The NMRProcFlow integration module allowed the quantification of several additional metabolites. The number of quantified metabolites and quantification trueness improved for some metabolites with deconvolution tools. Trueness and precision were not significantly different between zgpr- and NOESYpr-based spectra for about 70% of the variables.

**Conclusion:**

External calibration performed better than TSP internal referencing. Inter-laboratory tests are useful when choosing to better rationalize the choice of quantification tools for NMR-based metabolomic profiling and confirm the value of spectra deconvolution tools.

**Supplementary Information:**

The online version contains supplementary material available at 10.1007/s11306-023-02028-4.

## Introduction

The use of nuclear magnetic resonance spectroscopy (NMR) in metabolomics is increasing for large-scale biochemical phenotyping (Beckonert et al., 2007; Dona et al., 2014; Vignoli et al., 2019), especially for clinical and personalized medicine purposes (Letertre et al., 2021). NMR-based metabolomics provides reproducible robust fingerprinting with relative quantification of biological samples. However, the absolute quantification of individual metabolites in complex biological samples such as extracts and biofluids remains crucial in quantitative metabolomics with targeted metabolic profiling, especially for biomedical applications (Crook & Powers, 2020; Wishart, 2008; Wishart et al., 2022). Several strategies exist for quantitative metabolomics based on 1D or 2D NMR, in terms of sample preparation, data acquisition and data processing methods. For biofluid spectra acquisition, the NOESY (Nuclear Overhauser Effect SpectroscopY) pulse sequence with presaturation of the water signal (NOESYpr) is generally advised (Emwas et al., 2016). However, the simple presaturation-90° pulse-acquisition sequence (“zgpr” for Bruker and “proton with presaturation” for JEOL) is also largely used for relative [e.g. Snytnikova et al., 2019; Standage et al., 2021)] and absolute quantification [e.g. Camacho-Barcia et al., 2021; Melis et al., 2019; Monakhova et al., 2011)] owing to its accuracy, robustness and simplicity. The zgpr pulse sequence also allows an easier optimization of the power used to presaturate the water signal. Both zgpr and NOESYpr pulse sequences belong to the same class of solvent presaturation methods. They are the most widely used in quantitative NMR metabolomics and are among the most robust and accurate methods. Popular selective refocusing methods such as excitation-sculpting are not recommended in metabolomic studies, since they are less robust and induce a distortion of peak areas based on J-modulation (Giraudeau et al., 2015).

Spectra pre-processing steps that lead to Fourier-transformed, phased and baseline-corrected spectra usable for quantitative analysis can be performed with equipment-related software, commercial processing software or with open tools. For spectra processing steps that aim at extracting peak areas and calculating absolute concentrations, various approaches have been described. Although peak integration after peak picking is largely used, tools performing peak fitting with reference compound spectra acquired with the same standard operating procedure or spectral decomposition (often named ‘deconvolution’) are also available to deal with possible resonance overlapping. For instance, Chenomx NMR Analysis software (Chenomx, Edmonton, Canada) performs peak fitting, while Mnova (Mestrelab Research, Santiago de Compostela, Spain) and the MetaboDecon1D open package (Häckl et al., 2021) perform deconvolution. To determine absolute concentrations in the NMR tube, two strategies prevail: referencing with an internal standard (dissolved in the sample or more rarely as an external reference using a reference material contained in a separate solution) or calibration with dedicated solutions of commercial compounds (Cullen et al., 2013). For internal referencing, a reliable internal standard is required. Trimethylsilylpropionic-2,2,3,3-*d4* acid sodium salt (TSP or TMSP) or 3-(trimethylsilyl)-1-propanesulfonic 2,2,3,3,4,4-*d6* acid sodium salt (DSS), also used for chemical shift referencing in aqueous solutions (Wishart et al., 1995), may be used when macromolecules are not present in the sample. Otherwise macromolecules would interact with TSP (Shimizu et al., 1994). For external calibration, acquisitions must be performed in the exact same conditions as for the analysed mixture, including pH (Giraudeau et al., 2014). Interestingly, electronic reference methods exist (Akoka et al., 1999; Jung et al., 2016) but are not employed routinely in biofluid metabolomics. While there are many integration tools, reference methods and corresponding protocols available, there is no real consensus on the processing workflow to determine absolute metabolite concentrations.

**Fig. 1:**
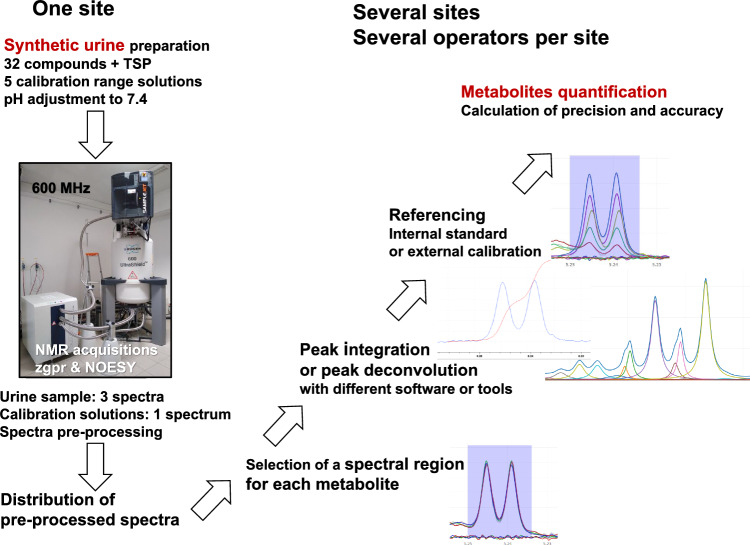
Experimental design of inter-laboratory test for metabolite quantification of synthetic urine

The objectives of the present study were to compare commonly used NMR software or tools using NMR peak integration or deconvolution, and to evaluate the operator or software effect on the trueness of quantitative results. For this study, we used 1D ^1^H-NMR spectra of a synthetic urine sample based on the experimental design described in Fig. 1. The use of a biofluid model sample is relevant since biological fluids such as urine are widely studied in metabolomics, and standard protocols have been proposed for the robust accurate quantification of potential urinary biomarkers by NMR (Emwas et al., 2016).

## Materials and methods

### Sample preparation

NMR tools were assessed using a homemade solution that imitates real urine (CDC-Center for Disease Control & Prevention, 2010), thereafter named ‘synthetic urine’ containing known amounts of 32 commercial metabolites. The metabolites and their concentrations were chosen from published data (Bouatra et al., 2013), and by selecting concentrations compatible with the sensitivity of proton NMR and metabolite signals spread over the full spectral width. Synthetic urine was prepared and pH-adjusted as detailed in Online Resource 1. Metabolite concentrations in the NMR tube ranged from 64 to 7959 µM (Online Resource 1). Five calibration range solutions containing the 32 compounds (0.1–12 mM concentrations) were also prepared (Online Resource 1). The synthetic urine and calibration solutions were supplemented with TSP for chemical shift calibration and 700 µl were transferred into a 5-mm NMR tube for a SampleJet autosampler (Bruker, Wissembourg, France).

### NMR acquisition and spectra pre-processing

An Avance-III HD Ultrashield 600-MHz spectrometer was used (Bruker BioSpin, Wissembourg, France), equipped with an ATMA CPQCI cryo-probe flushed with N_2_ with Z-gradient coils for 5-mm NMR tubes. The ^1^H-NMR spectra were acquired with a 1D pulse sequence with presaturation (Bruker “zgpr”), a 90° pulse angle, a 12-ppm spectral width, a 5-s acquisition time, a 35-s recovery delay, two dummy scans and 32 scans. The presaturation power level was adjusted to obtain an intensity of the residual water peak as intense as the most intense metabolite peak. NOESYpr spectra were acquired with a “noesypr1d” pulse sequence with a 12-ppm width, a 5-s acquisition time, a 35-s recovery delay, a 0.1-s mixing time, two dummy scans and 32 scans. The choice of a long recovery delay (35 s) for zgpr and NOESYpr spectra was based on T1 relaxation times of metabolites. For each pulse sequence, the 40-s total inter-scan resulting delay ensured full longitudinal relaxation for all ^1^H signals of interest. The receiver gain determined on the synthetic urine sample was used for all samples. The spectrum of the synthetic urine sample was acquired three times (without removing the NMR tube from the magnet) and that of each calibration range solution once. The spectra were pre-processed as follows: Fourier transformation, manual phase correction and automatic baseline correction with a polynomial of degree three, with TopSpin v4.0.7 (Bruker BioSpin, Karlsruhe, Germany) at one site by one operator and sent to the other sites. The spectra were deposited in the recherche.data.gouv.fr open repository (https://doi.org/10.57745/J0Y81K).

### Metabolite quantification

For the pre-processed zgpr spectra, each operator quantified the urinary metabolites with the TopSpin integration module (Bruker BioSpin, Karlsruhe, Germany), using either internal referencing with TSP or external calibration with the calibration-range solutions, as well as his/her favourite in-house tool or open-access or commercial NMR software (Online Resource 2). The integration regions for each metabolite were chosen by each operator independently focussing on non-overlapping peaks whenever possible and are indicated in Online Resources 3–4 and at https://doi.org/10.57745/J0Y81K.

#### Quantification using TopSpin integration module and internal referencing with TSP

Each operator chose one signal per metabolite and integrated it using the TopSpin integration module (Bruker BioSpin, Karlsruhe, Germany). The metabolite concentration was then calculated according to expression (1).

1 $$Cx = \frac{{Ix \times Cs \times Ns{ }}}{Is \times Nx}$$

*Cx* is the metabolite concentration, *Ix* the integral of the metabolite peak, *Nx* the number of protons contributing to the signal, *Cs* the standard concentration, *Is* the integral of the standard proton signal, and *Ns* the number of protons contributing to the standard proton signal.

#### Quantification using the TopSpin integration module with calibration curves

For each metabolite, a calibration curve (*Ix* = *a* × *Cx* + *b*) was plotted using the integrated areas of a selected metabolite peak in each calibration-range solution spectrum as a function of the corresponding metabolite concentrations. The curve was fitted with a linear regression model. Metabolite concentrations in the synthetic urine were then determined by incorporating peak integral values into the corresponding regression model. Each operator chose independently one signal per metabolite and used the same integration regions for the urine solution and the calibration-range solutions as for the TSP referencing method.

#### Quantification using the NMRProcFlow integration module with calibration curves

One operator used the NMRProFlow online tool [https://nmrprocflow.org/, (Jacob et al., 2017)] for local peak realignment of the received 1D spectra, for the determination of the peak integration regions and for concentration calculations based on the five calibration-range solutions.

#### Quantification using Mnova deconvolution tool

One operator used MestReNova software (Mnova, Mestrelab Research, Santiago de Compostela, Spain) with the Simple Mixture Analysis plugin for semi-automatic metabolite quantification, using the received 1D spectra. This software was used to define the integration regions as peaks or multiplets, using a signal deconvolution tool (GSD) to extract the integrals and thus quantify the concentrations of the identified metabolites. The synthetic urine concentration measurements were based on a library of spectra of the calibration-range solutions and their concentrations.

#### Quantification using MetaboHUB in-house deconvolution tool “NMRDeconvR”

One operator used an in-house application based on a dedicated R package (https://github.com/INRA/Rnmr1D), hereafter named “NMRDeconvR”. Its main functions are noise reduction, baseline correction, automatic ppm calibration based on TSP or DSS and deconvolution. The latter is based on the search for peaks from the second derivative of the signal, then on the construction of a spectrum modelled as a sum of Voigt pseudo-function shapes, followed by an optimisation step based on the Levenberg–Marquardt algorithm (Levenberg 1944). In the present experiment, only local baseline correction and deconvolution were used. Quantification was performed using Jupyter Notebooks (Kluyver et al., 2016), a useful tool for collaborative open data (Mendez et al., 2019). A first notebook was used to test the deconvolution of each of the zones corresponding to the targeted metabolites to optimise the parameters. This set of parameters was then compiled in a parameter file. A second notebook focused on the deconvolution and quantification of the zones corresponding to the targeted metabolites in all spectra, the calibration-range spectra being used for calibration.

For the pre-processed NOESYpr spectra, the best quantification method with TopSpin integration was used by five operators and the best deconvolution method was used by one operator.

### Statistical analyses

A principal component analysis (PCA) of the synthetic urine data was performed on mean-centred and unit-variance scaled data using R scripts [biostatflow.org, v2.9, (Jacob et al., 2020)]. The theoretical synthetic urine sample—obtained based on gravimetric concentrations—was added on the scores plot. Precision (coefficient of variation expressed as a percentage, n = 3 spectra) and relative trueness (ratio of the mean measured value based on the three urine spectra minus the theoretical value over the theoretical value, expressed as a percentage) values were calculated for each metabolite quantified by each operator based on his/her data obtained using the three zgpr or NOESYpr pre-processed spectra. Mean and standard deviations were calculated for the spectra processing strategies performed by several operators. A Kruskal–Wallis analysis (*P* < 0.05) was performed using jamovi [v2.2, (Jamovi Team, 2021)] to study the operator effect for the external calibration strategy performed on the zgpr spectra. NOESYpr- and zgpr-based precisions and trueness of metabolite quantifications were compared using Wilcoxon tests performed with biostatflow.org (*P* < 0.05).

## Results

This study involving several sites and operators evaluated the impact of processing and quantification software and methods. Several operators performed the same two protocols for peak integration with TopSpin followed by TSP referencing (zgpr spectra) or external calibration (zgpr and NOESYpr spectra). For these protocols, the peak integration boundaries were purposely operator-dependent. NMRProcFlow and the two deconvolution tools were used by a single operator each. Quantification results were first analysed separately for each type of spectra and then compared.

### Spectra observation and annotation

**Fig. 2:**
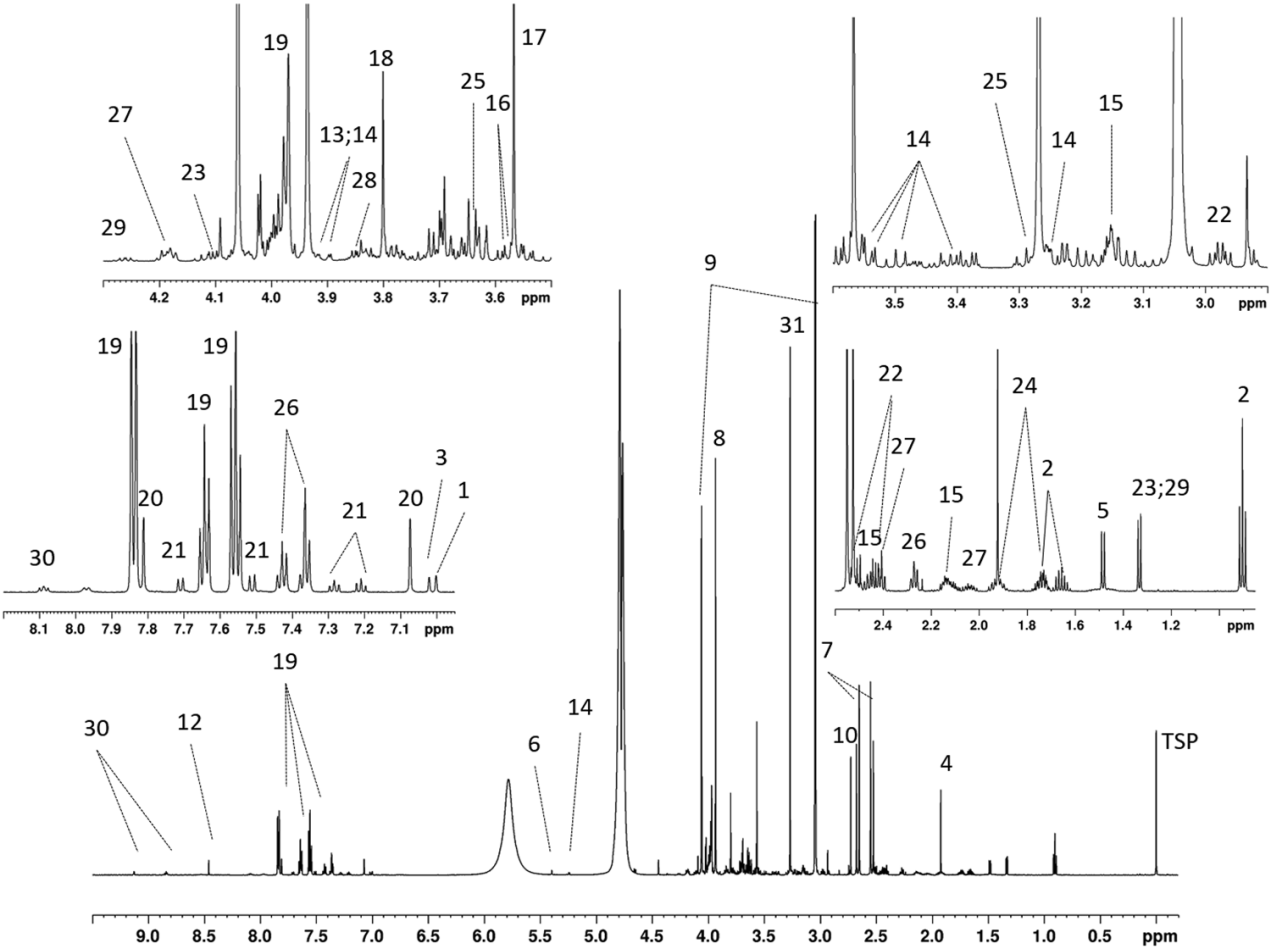
Representative 1D ^1^H-NMR spectrum of synthetic urine with metabolite annotation: zgpr spectrum. Numbers indicate the following metabolites: 1: 1-methylhistidine; 2: 2-hydroxybutyric acid; 3: 3-methylhistidine; 4: acetic acid; 5: alanine; 6: allantoin; 7: citric acid; 8: creatine; 9: creatinine; 10: dimethylamine; 11: ethanolamine; 12: formic acid; 13: fructose; 14: glucose; 15: glutamine; 16: glycerol; 17: glycine; 18: guanidoacetic acid; 19: hippuric acid; 20: histidine; 21: indoxylsulfate; 22: isocitric acid; 23: lactic acid; 24: lysine; 25: myo-inositol; 26: phenylacetylglutamine; 27: pyroglutamic acid; 28: serine; 29: threonine; 30: trigonelline; 31: trimethylamine-N-oxide

The synthetic urine spectra contained about 410–420 detectable (peak picking with TopSpin software) and 350–360 quantifiable (i.e. with a signal-to-noise ratio over 10) peaks for the zgpr and NOESYpr spectra. The quality of the baseline correction was similar in both spectra. The quantifiable peaks in the zgpr urine spectra were 1.1 to 1.3 times more intense than those in the NOESYpr spectra (Online Resource 5). The synthetic urine zgpr and NOESYpr spectra were annotated by each operator based on a comparison with the proton NMR spectra of pure compounds recorded using the same experimental parameters (Fig. 2, Online Resource 6). An interactive annotated zgpr-spectrum is available at http://pmb-bordeaux.fr/nmrAnnot/urine.html. The four most intense signals corresponded to creatinine, trimethylamine-N-oxide, creatine and citric acid.

**Fig. 3:**
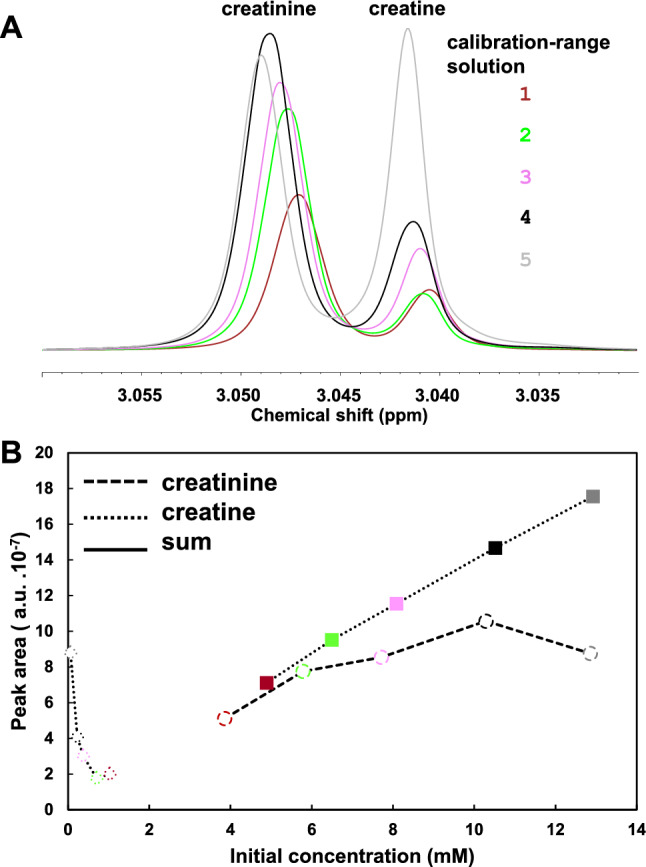
Creatine and creatinine resonances in zgpr spectra and peak areas for five calibration range solutions. **A** Zoom-in on creatinine and creatine spectra region. **B** Plot of corresponding peak areas as a function of concentrations in synthetic urine solution. Ordinates: peak areas of creatine (open diamonds) and creatinine (open circles) resonances determined by one operator using peak integration with TopSpin (PI1), and of their sum (full squares). Abscissa: theoretical concentrations in synthetic urine at time of its preparation, i.e. “initial concentration”. *a.u. *arbitrary units

Creatine and creatinine could not be quantified individually in the zgpr or NOESYpr spectra owing to the instability of creatine and a complex equilibrium between these two compounds (Fig. 3 for zgpr). The areas of creatine or creatinine peaks in the 3.03–3.06 spectra region were not proportional to their initial theoretical individual concentrations in the calibration-range solutions. However, their sum was proportional to the initial ‘creatinine + creatine’ concentration, which allowed the quantification of the total ‘creatinine + creatine’ concentration with all spectra processing strategies. Since lactic acid and threonine could not be quantified individually in the zgpr or NOESYpr spectra using peak integration owing to peak overlapping, their sum was quantified. Their individual quantification was tested using deconvolution. Cysteine in solution is known to be unstable over time (Krattenmacher et al., 2019; Zecchini et al., 2019) and especially at pH 7.4 as mentioned previously (Krattenmacher et al., 2019; Zecchini et al., 2019): it was dimerized to cystine that was used to estimate it.

### Quantifications based on zgpr spectra

**Fig. 4:**
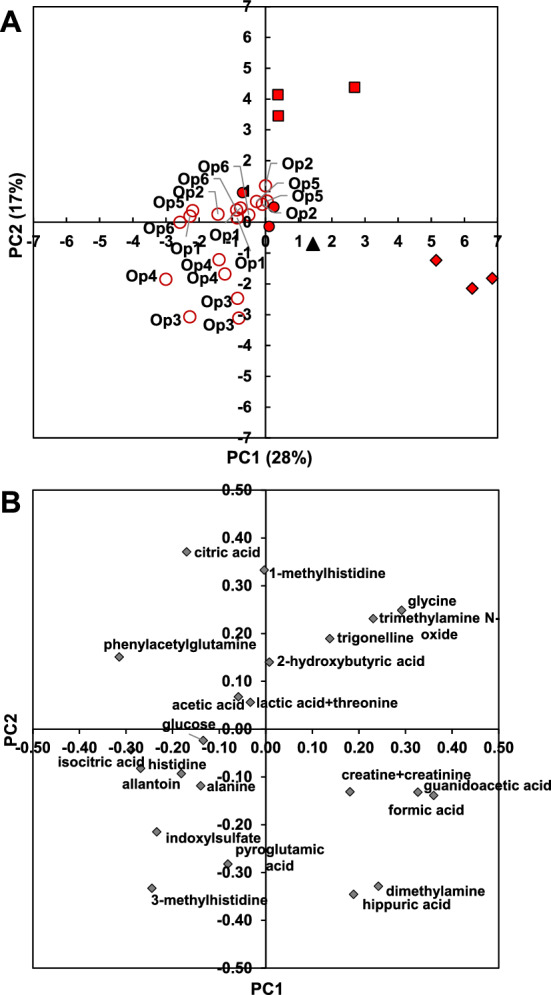
PCA of quantification data of 20 metabolites and two sums of two metabolites of synthetic urine quantified in zgpr spectra with external calibration. **A** Scores plot. Open circle, peak integration with TopSpin (PI1-ExtCal); closed circle, peak integration with NMRProcFlow (PI2-ExtCal); diamond, deconvolution with Mnova (DC1-ExtCal); square, deconvolution with NMRDeconvR (DC2-ExtCal); triangle, plot of theoretical sample. For peak integration with TopSpin performed by six operators, operator is annotated. **B** Loadings plot with metabolite annotation

The spectral regions selected for peak integration (PI1 and PI2) or deconvolution (DC2) are listed in Online Resource 3 and available at https://doi.org/10.57745/J0Y81K, respectively. For most metabolites, the operators chose the same spectral regions. Some metabolites were not quantifiable by all methods, commercial or in-house NMR software or tools (Online Resource 7). Twenty metabolites and the sums of creatine and creatinine or lactic acid and threonine were successfully quantified using all quantification strategies (Table 1). To obtain an overview of these quantification data, a PCA was performed on these 22 concentrations (Online Resource 8). The ‘theoretical’ sample, based on gravimetric metabolite concentrations, was plotted in the PC1 × PC2 plane using the loadings values of the PCA performed without it. The first principal component (PC1, 46% of total variability) separated the processing strategy based on internal referencing with TSP from all the strategies using external calibration. The quantifications based on external calibration were much closer to the theoretical sample. Comparison of the scores and loadings plots (Online Resource 8) showed that internal referencing tended to over-estimate several metabolites, including 2-hydroxybutyric, acetic, formic and isocitric acids, alanine, histidine, phenylacetylglutamine and trigonelline. A PCA was then performed on the 20 common metabolites or metabolite sums quantified using external calibration only (Fig. 4), which revealed three sample groups in the PC1 × PC2 plane. PC1 (28% of total variability) separated the Mnova-based quantifications using deconvolution from the TopSpin-based and NMRProcFlow-based quantifications. NMRDeconvR tool quantifications were intermediary. PC2 separated the NMRDeconvR-based quantifications from all other quantification data. Comparison of the scores plot (Fig. 4A) and the loadings plot (Fig. 4B) showed that the quantification of formic acid, guanidoacetic acid and the sum of creatine and creatinine tended to be higher with Mnova, and the quantification of citric acid and 1-methylhistidine tended to be higher with the NMRDeconvR than with TopSpin. The plotted theoretical sample was intermediary between the three sample groups. These tendencies were verified and detailed using precision and trueness calculations.

**Table 1:** Precision for 20 metabolites and two sums of metabolites quantified from synthetic urine zgpr spectra using all strategies for resonance integration and calibration, and for metabolites quantified using certain strategies only

	Precision (CV%)						
Quantification strategy	PI1-TSPref		PI1-ExtCal		PI2-ExtCal	DC1-ExtCal	DC2-ExtCal
Metabolite	Mean value (n = 6 op.)	SD	Mean value (n = 6 op.)	SD	Value (n = 1 op.)	Value (n = 1 op.)	Value (n = 1 op.)
1-methylhistidine	2.37	0.67	2.96	0.80	1.07	3.78	4.75
2-hydroxybutyric acid	0.28	0.07	0.69	0.16	0.59	0.58	1.12
3-methylhistidine	1.49	0.28	1.65	0.30	1.14	3.13	2.73
Acetic acid	0.15	0.08	0.26	0.02	0.78	1.08	1.53
Alanine	0.41	0.09	0.57	0.13	0.27	2.12	1.37
Allantoin	13.72	3.17	34.47	7.37	4.72	2.80	4.19
Citric acid	0.31	0.07	0.10	0.00	0.03	0.10	1.00
Cysteine^a^	ND		ND		5.11	ND	3.64
Dimethylamine	0.45	0.14	0.22	0.07	0.20	1.40	1.33
Ethanolamine	ND		ND		0.63	ND	2.23
Formic acid	0.54	0.34	0.33	0.17	0.74	0.88	1.68
Fructose					ND	ND	7.20
Glucose	18.30	8.58	32.76	14.51	3.05	7.34	0.98
Glutamine	ND		ND		2.43	ND	3.73
Glycerol	ND		ND		0.20	ND	2.61
Glycine	0.49	0.13	0.26	0.07	0.34	0.59	2.44
Guanidoacetic acid	0.54	0.07	0.33	0.04	0.13	1.44	3.95
Hippuric acid	0.17	0.08	0.19	0.02	1.13	1.40	0.82
Histidine	0.29	0.03	0.62	0.09	0.26	1.35	2.34
Indoxylsulfate	2.90	0.71	3.29	0.45	0.31	1.89	3.77
Isocitric acid	1.22	0.28	1.11	0.18	0.56	0.81	4.99
Lactic acid	ND		ND		9.09	ND	8.08
Lysine	ND		ND		4.59	ND	7.34
Myo-inositol	ND		ND		1.54	ND	12.37
Phenylacetylglutamine	0.62	0.17	0.72	0.07	0.58	3.64	0.97
Pyroglutamic acid	1.68	0.60	2.48	1.03	3.47	1.83	3.10
Serine	ND		ND		ND	ND	3.36
Threonine	ND		ND		1.81	9.84	5.93
Trigonelline	0.83	0.09	0.67	0.19	1.02	0.55	1.66
Trimethylamine N-oxide	0.21	0.07	0.19	0.01	0.28	0.15	1.42
Creatine + creatinine	3.02	0.14	3.08	0.01	0.10	1.18	2.63
Lactic acid + threonine	5.69	8.85	1.11	1.52	0.16	0.69	0.89

Codes of quantification strategies are given in Online Resource 2

*op.* operator, *SD* standard deviation, *ND* not determined due to lack of quantifiable resonance

^a^Estimated from cystine

The corresponding precisions (Table 1) and relative trueness (Table 2) were calculated for all processing methods. For internal calibration with TSP, only about one half of the metabolites were quantified with an absolute value of trueness lower, i.e. better, than 5%. Among the metabolites with an absolute trueness above 5%, glucose and pyroglutamate were the most underestimated, and 2-hydroxybutyric acid and formic acid were the most overestimated (Table 2). Glucose was clearly impacted by residual water presaturation. Several resonances were impacted by peak overlap, e.g. the singlet at 1.92 ppm for acetic acid with resonances of the lysine multiplet at 1.97–1.87 ppm, resulting in impaired quantification of lysine for several operators and a slight overestimation of acetic acid quantification for all operators. However, with TopSpin integration module and external calibration, about 90% of the metabolites and metabolite sums were quantified with an absolute trueness better than 5%. The quantification trueness improved for several metabolites with deconvolution, either with Mnova or NMRDeconvR and external calibration (all 20 individual metabolites common to all strategies quantified with an absolute value of trueness better than 5%).

**Table 2:** Relative trueness for 20 metabolites and two sums of metabolites quantified from synthetic urine zgpr spectra using all strategies for resonance integration and calibration, and for metabolites quantified using certain strategies only

	Relative trueness (%)						
Quantification strategy	PI1-TSPref		PI1-ExtCal		PI2-ExtCal	DC1-ExtCal	DC2-ExtCal
Metabolite	Mean value (n = 6 op.)	SD	Mean value (n = 6 op.)	SD	Value (n = 1 op.)	Value (n = 1 op.)	Value (n = 1 op.)
1-methylhistidine	−0.96	0.94	−2.50	6.01	−0.34	−2.72	3.40
2-hydroxybutyric acid	18.44	2.11	3.60	0.07	5.29	3.58	4.16
3-methylhistidine	−4.92	0.56	4.46	4.27	0.46	−3.00	−2.91
Acetic acid	16.14	2.40	2.68	0.04	16.45	0.10	2.71
Alanine	13.28	3.95	1.06	0.31	0.56	−0.01	0.02
Allantoin	12.41	10.26	13.77	1.16	11.34	4.95	2.02
Citric acid	−6.43	2.50	1.37	0.02	1.46	−1.02	3.80
Cysteine^a^	ND		ND		6.77	ND	1.02
Dimethylamine	−3.88	2.76	0.84	1.27	0.27	4.59	0.07
Ethanolamine	ND		ND		−4.48	ND	1.70
Formic acid	21.33	1.27	−2.19	0.20	−2.51	2.61	−1.88
Fructose	ND		ND		ND	ND	20.53
Glucose	−36.83	46.44	−0.47	5.05	0.14	−0.06	−2.49
Glutamine	ND		ND		3.25	ND	0.29
Glycerol	ND		ND		−3.39	ND	−4.08
Glycine	1.79	2.82	−1.46	1.04	−1.16	2.56	2.70
Guanidoacetic acid	−2.50	5.86	−1.49	0.45	−0.72	3.66	−1.36
Hippuric acid	−1.30	39.40	0.57	0.48	1.39	2.60	−1.09
Histidine	9.00	0.97	3.19	0.85	2.43	0.68	1.23
Indoxylsulfate	5.21	2.00	2.02	1.24	0.78	−2.25	−3.32
Isocitric acid	13.32	1.74	5.38	0.46	0.14	−2.45	1.65
Lactic acid	ND		ND		5.46	ND	10.40
Lysine	ND		ND		−14.31	ND	2.67
Myo-inositol	ND		ND		−6.96	ND	−9.74
Phenylacetylglutamine	7.21	1.72	1.50	0.04	1.58	−4.16	1.79
Pyroglutamic acid	−49.62	117.24	−0.12	5.97	−2.00	−3.52	−2.70
Serine	ND		ND		ND	ND	5.07
Threonine	ND		ND		−2.64	0.97	−3.54
Trigonelline	6.26	2.13	−0.28	0.17	−1.40	−0.03	1.18
Trimethylamine N-oxide	−1.94	2.53	1.47	0.23	1.56	2.41	2.92
Creatine + creatinine	−6.26	2.62	−2.28	0.25	−0.51	1.96	−3.19
Lactic + threonine	6.82	9.29	6.31	9.22	1.67	1.06	3.51

Codes of quantification strategies are given in Online Resource 2

*op*. operator, *SD* standard deviation, *ND* not determined due to lack of quantifiable resonance

^a^Estimated from cystine

The most accurate quantification strategy performed by several operators was peak integration with TopSpin followed by external calibration. For this strategy, we compared the variability between the operators for all 22 concentrations. Based on a Kruskal–Wallis test (*P* < 0.05, Online Resource 9), the operator effect was significant for six out of the 22 variables: 3-methylhistidine, glycine, guanidoacetic acid, hippuric acid, pyroglutamic acid, and lactic acid plus threonine. For hippuric acid and pyroglutamic acid, this effect is due to the fact that the resonances selected for integration differed between operators. For glycine, this effect may be due to partial peak overlapping with glycerol.

In addition to these 22 concentrations, threonine was quantified individually using deconvolution with Mnova (Online Resource 7). All these metabolites, as well as cysteine, ethanolamine, glutamine, glycerol, lactic acid, lysine and myo-inositol were quantified using NMRProcFlow and external calibration (Online Resource 7). However, only ethanolamine, glutamine and glycerol had an absolute value of trueness better than 5%. With NMRDeconvR, cystine and lysine also had an absolute trueness better than 5% (Table 2). NRMDeconvR allowed the quantification of serine (3.4% precision and 5.1% trueness) and the estimation of fructose using the difference between fructose plus glucose and glucose (7.2% precision and 20.5% trueness).

### Quantifications based on NOESYpr spectra

For NOESYpr, the best two quantification methods determined from zgpr data were evaluated, i.e. TopSpin integration and NMRDeconvR, both used with external calibration. Twenty-one metabolites and the sums of creatine and creatinine or lactic acid and threonine were successfully quantified from NOESYpr spectra using these quantification strategies. The precision (Online Resource 10) and trueness (Online Resource 11) values were calculated for the quantification data obtained from peak integration by several operators (23 concentrations) or from deconvolution by one operator (30 concentrations). For peak integration, 21 concentrations were quantified with a precision better than 5%, and among the latter, 17 metabolites were quantified with an absolute value of trueness better than 5%. For peak deconvolution, ethanolamine, glutamine, glycerol and lysine, which could not be quantified with peak integration, were quantified with a precision and an absolute value of trueness better than 5%.

For peak integration which was performed by several operators, the precision and trueness values were compared to the corresponding values obtained by the same operators using the zgpr spectra (Table 3, Wilcoxon test, *P* < 0.05). Precision was not significantly different between zgpr- and NOESYpr-based spectra for 15 out of the 22 variables. It was significantly lower, i.e. better for 2-hydroxybutyric acid, alanine, phenylacetylglutamine and creatine plus creatinine in NOESYpr-based data, and for formic acid, isocitric acid and trimethylamine-N-oxide in zgpr-based data. Trueness was not significantly different between zgpr- and NOESYpr-based spectra for 16 out of the 22 variables. It was significantly better for acetic acid, allantoin, isocitric acid and phenylacetylglutamine in NOESYpr-based data, and for formic acid and trimethylamine-N-oxide in zgpr-based data.

**Table 3:** Comparison of precisions and relative trueness on concentration of 20 metabolites and two sums of metabolites quantified from zgpr and NOESYpr spectra recorded on synthetic urine and processed using TopSpin integration and external calibration (PI1-ExtCal) by five operators

Metabolite	NOESYpr CV over zgpr CV	Wilcoxon test p-value	NOESYpr trueness over zgpr trueness	Wilcoxon test p-value
1-methylhistidine	0.816	1.508E−01	−94.773	6.905E−01
2-hydroxybutyric acid	0.311	1.587E−02	0.523	1.000E+00
3-methylhistidine	0.789	9.524E−02	1.780	5.476E−01
Acetic acid	0.959	1.508E−01	0.439	7.937E−03
Alanine	0.450	7.937E−03	0.159	9.524E−02
Allantoin	0.978	8.413E−01	0.403	7.937E−03
Citric acid	1.002	1.508E−01	−3.369	9.524E−02
Dimethylamine	1.258	1.508E−01	4.496	8.413E−01
Formic acid	3.572	7.937E−03	−1.504	7.937E−03
Glucose	1.452	2.222E−01	−2.289	9.524E−02
Glycine	0.833	8.413E−01	1.538	1.000E+00
Guanidoacetic acid	1.376	1.508E−01	3.395	1.000E+00
Hippuric acid	1.138	6.905E−01	1.766	4.206E−01
Histidine	1.242	5.476E−01	0.726	6.905E−01
Indoxylsulfate	0.797	5.556E−02	−0.034	9.524E−02
Isocitric acid	1.386	7.937E−03	−0.801	7.937E−03
Phenylacetylglutamine	0.688	7.937E−03	0.354	7.937E−03
Pyroglutamic acid	1.197	1.508E−01	−0.508	5.556E−02
Trigonelline	1.920	5.556E−02	−0.703	2.222E−01
Trimethylamine-N-oxide	1.255	7.937E−03	3.404	7.937E−03
Creatine + creatinine	0.979	7.937E−03	1.452	3.095E−01
Lactic acid + threonine	0.864	4.206E−01	1.403	6.905E−01

Ratios between NOESYpr- and zgpr-based precision or trueness and p-value of corresponding Wilcoxon tests

## Discussion

### Although the synthetic urine contained no macromolecules, TSP referencing was not satisfactory for metabolite quantification

In the present experiment, we used a buffer solution and adjusted the pH of all samples, and had similar ionic strengths in the synthetic urine and the calibration-range solutions, to limit uncontrolled chemical shift variations. As no protein or lipid that may interact with TSP was present in the synthetic urine, no global overestimation of metabolite concentrations was expected. Glucose concentration was largely underestimated when using TSP referencing, which is in line with an impact of residual water presaturation on regions close to the water signal (Giraudeau et al., 2015). Pyroglutamate underestimation was probably due to peak overlapping. The eight metabolites overestimated by TSP referencing corresponded to low- or medium-intensity resonances below 2.3 ppm or above 7.3 ppm.

To avoid possible biases with TSP added in the samples for NMR-based urine analyses, alternatives have been suggested in the literature, such as the use of alternative internal references [sodium acetate or sodium formate, (Emwas et al., 2018)] or the use of external standards (Crook & Powers, 2020). While there is no consensus on the choice of a chemical reference, our results point to the need to carefully analyse quantification results obtained with such methods.

### During our experiment, all metabolites were stable except cysteine, creatinine and creatine

In the synthetic urine and in the calibration solutions, cysteine was dimerized into cystine that was used to estimate it. Therefore, in a real urine sample, the sum of cysteine and cystine could be estimated. Since the total creatinine output in urine is considered constant and creatinine seems quite stable during sample storage, many investigators normalize their results to the creatinine content (Spierto et al., 1997). However, in the present experiment, creatine appeared especially unstable in the calibration solutions. Stability issues concerning storage have previously been reported for urine samples for several metabolites including creatinine (Saude & Sykes, 2007). As observed in the present experiment, the increased concentration of creatine and the instability of creatinine are in line with previous findings on urine using NMR analyses to study storage techniques (Saude & Sykes, 2007). This instability was suggested to be of bacterial origin, but temperature- and pH-dependent non-enzymatic reactions were also mentioned in vitro (Wyss & Kaddurah-Daouk, 2000). For fingerprinting, normalization of urine contents to creatinine level must thus be used with caution. An alternative physiological normalization to the sum of creatinine and creatine seems a possibility when no significant dysregulation of metabolism is expected. Normalization using osmolality or specific gravity is also an option (Emwas et al., 2018).

### Operator effect on quantification was limited but could be controlled better

For the processing protocol followed by the largest number of operators on zgpr spectra, i.e. peak integration with TopSpin followed by external calibration, an operator effect was observed for five metabolites and one sum of metabolites only. This effect can be accounted for by a different choice of integrated resonance, as it is more difficult to define integration boundaries for some peaks than for others owing to peak overlapping. This is particularly true for lactic acid and threonine and for low-intensity and complex patterns such as pyroglutamic acid. This operator effect could also be explained by the lower peak intensities for some signals. Indeed, in a previous experiment using synthetic urines with variable-size bucketing, CV and SNR were shown to have a weak but clear inverse relationship (Wang et al., 2013). Training of newcomers by expert users and a clear definition of criteria for selecting resonances and peak boundaries could limit this effect when using TopSpin. Semi-automatic definition modes of peak boundaries based on adaptative binning (Anderson et al., 2011; De Meyer et al., 2008) or on recent methods of deconvolution (Li et al., 2023; Schmid et al., 2023) could also reduce or avoid such operator-dependent effects.

### Tailored processing tools improved quantification with external calibration

With external calibration, ethanolamine, glutamine, glycerol and threonine were accurately quantified using NMRProcFlow, unlike with TopSpin. With NMRProcFlow, it is possible to interactively perform small local realignments of resonances and select a resonance from a complex group before integration, e.g. realignment and selection of one resonance for a triplet as done for ethanolamine. Likewise, the NMRDeconvR tool made it possible to quantify the metabolites listed above as well as cysteine, and to estimate fructose which could not be quantified with any other strategy. Fructose and cysteine were at low concentrations in the initial mixture, with most NMR signals appearing as multiplets and with chemical shifts in spectral regions with ubiquitous peak overlapping. Therefore, deconvolution with manual adjustment of the peak ranges is key to extracting corresponding peak areas. This was not achieved with the Mnova tool here since the peak range was not manually readjusted by the operator.

Overall, for quantification with external calibration, trueness was improved for nearly all variables and for all strategies when using NMRProcFlow or NMRDeconvR, and for most of them when using Mnova. This result was expected for deconvolution, as peak overlapping concerned most of the metabolites present in the synthetic urine solution. This is in line with previous comparisons of 1D-spectra deconvolution with other automated approaches for identification and quantification of metabolites using spiked urine or plasma samples (Zheng et al., 2011). Precision was improved for at least half or about half of the variables with NMRProcFlow or Mnova, respectively. However, our deconvolution approaches using external calibration may somehow be penalized by the distortion of certain peaks of the calibration spectra, unlike NMRProcFlow for which the approach by integration of identical zones for both synthetic urine and calibration spectra tolerates peak deformation very well.

### Compared to zgpr, NOESYpr had advantages and drawbacks

1D NOESYpr pulse sequences have been advised for NMR-based metabolomics (Mckay, 2011), especially for biofluids as they provide an efficient suppression of the faraway water magnetization (Giraudeau et al., 2015), although the mixing time sometimes needs to be optimised. In the present experiment, trueness and precision were not significantly different between zgpr- and NOESYpr-based spectra for about 70% of the variables. This is in line with a study on cerebrospinal fluid showing that the metabolite concentrations obtained using DSS internal referencing and zgpr and noesypr1d, or noesygppr1d and zgpr sequences, were similar (Kolokolova et al., 2010). In the present experiment, trueness was slightly yet significantly better for four variables in the NOESYpr-based data, and for two variables in the zgpr-based data. Precision was slightly yet significantly better for four variables in the NOESYpr-based data, and for three variables in the zgpr-based data. Overall, the choice of the optimal pulse sequence is dependent on the study, matrix and operator. Most importantly, our conclusions on the choice of the processing and integration approach remain valid, regardless of the pulse sequence used.

### Transferability to real urine samples

In the present study, we used a synthetic urine with known metabolite concentrations to be able to calculate trueness values for metabolite quantifications. However, most of our conclusions based on this synthetic solution mimicking urine should apply to real urine samples.

Irrespective of the spectra processing method chosen, the adjustment of some acquisition parameters will be needed, since presaturation parameters are sample dependent, and their optimization is key to avoid or limit effects on signals close to the residual water peak. When transferring the methods to real urine samples, the presaturation power level will need to be adjusted so that the intensity of the residual water peak does not exceed the intensity of the most intense metabolite peaks, while avoiding using unnecessarily high power that would be detrimental to the quantification of nearby metabolite peaks.

For spectra processing, the fact that real urine samples contain peptides and proteins potentially interacting with TSP (Shimizu et al., 1994) reinforces the importance of external calibration compared to TSP referencing. In addition, an increased metabolite complexity [over 200 quantifiable metabolites in real urine (Bouatra et al., 2013)] and the presence of overlapping background signals from urinary peptides and small proteins may further bias the concentration values of several metabolites as shown for alanine (Gronwald et al., 2008). This increased spectral complexity points in favour of deconvolution tools. Moreover, in a set of real urine samples, differences of ionic strength among samples may result in uncontrolled chemical shift variations. To facilitate spectra processing, the ionic strength of the calibration solutions should be as close as possible to that of real urine, and realignment during the processing of spectra of calibration solutions and samples could be even more crucial than for synthetic urine. An experimental way to verify whether our results apply to real urine samples would be to use a spike-in experiment of a urinary biological sample (Klein et al., 2013).

## Conclusion

The present results highlight the relevance of inter-operator or inter-laboratory tests to better rationalize the choice of quantification tools in targeted NMR metabolomics and confirm the relevance of local 1D spectrum deconvolution to improve the trueness and precision of quantitative data. However, when 5% precision and trueness are sufficient, TopSpin integration with external calibration is of interest. Results also show that the use of TSP as an internal reference for quantitative analysis of urine is probably not the optimal choice, in line with recent results obtained on plasma that suggested the use of alternative reference compounds (Nagana Gowda et al., 2021). More accurate results can be obtained with an external calibration strategy, although this involves a heavier and more time-consuming experimental procedure. Finally, this study highlights the importance of inter-laboratory studies in the development of reliable processing methods to obtain accurate quantitative data in NMR metabolomics. Here, as a first step, we used a single data set and different operators to assess multiple processing software. Further steps would require complementary experimental designs. Using several NMR datasets analysed by a single operator with all the processing methods and associated software used in the present study would provide a more robust comparison of these processing methods. Using a set of identical samples distributed to several laboratories instructed to use a single acquisition and quantification method to quantify metabolites would provide a measure of inter-laboratory variability.

## Supplementary Information

Below is the link to the electronic supplementary material.

Supplementary file1 (PDF 574 KB)

## Data Availability

NMR spectra and their metadata have been deposited in data.gouv.fr repository, https://doi.org/10.57745/J0Y81K. An interactive annotated zgpr spectrum is available at http://pmb-bordeaux.fr/nmrAnnot/urine.html.

## Software availability

NMRDeconvR tool is available at https://github.com/INRA/Rnmr1D.

## References

[CR1] Akoka S, Barantin L, Trierweiler M (1999). Concentration measurement by proton NMR using the ERETIC method. Analytical Chemistry.

[CR2] Anderson PE, Mahle DA, Doom TE, Reo NV, DelRaso NJ, Raymer ML (2011). Dynamic adaptive binning: An improved quantification technique for NMR spectroscopic data. Metabolomics.

[CR3] Beckonert O, Keun HC, Ebbels TMD, Bundy J, Holmes E, Lindon JC, Nicholson JK (2007). Metabolic profiling, metabolomic and metabonomic procedures for NMR spectroscopy of urine, plasma, serum and tissue extracts. Nature Protocols.

[CR4] Bouatra, S., Aziat, F., Mandal, R., Guo, A. C., Wilson, M. R., Knox, C., Bjorndahl, T. C., Krishnamurthy, R., Saleem, F., Liu, P., Dame, Z. T., Poelzer, J., Huynh, J., Yallou, F. S., Psychogios, N., Dong, E., Bogumil, R., Roehring, C., & Wishart, D. S. (2013). The human urine metabolome. *PLOS ONE, *8(9), e73076. doi:10.1371/journal.pone.0073076. 10.1371/journal.pone.0073076 PMC3762851 24023812

[CR5] Camacho-Barcia L, García-Gavilán J, Papandreou C, Hansen TT, Harrold JA, Finlayson G, Blundell JE, Sjödin A, Halford JCG, Bulló M (2021). Circulating metabolites associated with postprandial satiety in overweight/obese participants: The SATIN study. Nutrients.

[CR6] CDC-Center for Disease Control and Prevention. (2010). Bisphenol A and other environmental phenols and parabens in urine. https://www.cdc.gov/nchs/data/nhanes/nhanes_07_08/eph_e_met_phenols_parabens.pdf.

[CR7] Crook AA, Powers R (2020). Quantitative NMR-based biomedical metabolomics: Current status and applications. Molecules.

[CR8] Cullen CH, Ray GJ, Szabo CM (2013). A comparison of quantitative nuclear magnetic resonance methods: Internal, external, and electronic referencing. Magnetic Resonance in Chemistry.

[CR9] De Meyer T, Sinnaeve D, Van Gasse B, Tsiporkova E, Rietzschel ER, De Buyzere ML, Gillebert TC, Bekaert S, Martins JC, Van Criekinge W (2008). NMR-based characterization of metabolic alterations in hypertension using an adaptive, intelligent binning algorithm. Analytical Chemistry.

[CR10] Dona AC, Jiménez B, Schäfer H, Humpfer E, Spraul M, Lewis MR, Pearce JTM, Holmes E, Lindon JC, Nicholson JK (2014). Precision high-throughput proton NMR spectroscopy of human urine, serum, and plasma for large-scale metabolic phenotyping. Analytical Chemistry.

[CR11] Emwas A-H, Roy R, McKay RT, Ryan D, Brennan L, Tenori L, Luchinat C, Gao X, Zeri AC, Gowda GAN, Raftery D, Steinbeck C, Salek RM, Wishart DS (2016). Recommendations and standardization of biomarker quantification using NMR-based metabolomics with particular focus on urinary analysis. Journal of Proteome Research.

[CR12] Emwas A-H, Saccenti E, Gao X, McKay RT, dos Santos VAPM, Roy R, Wishart DS (2018). Recommended strategies for spectral processing and post-processing of 1D 1H-NMR data of biofluids with a particular focus on urine. Metabolomics.

[CR13] Giraudeau P, Tea I, Remaud GS, Akoka S (2014). Reference and normalization methods: Essential tools for the intercomparison of NMR spectra. Journal of Pharmaceutical and Biomedical Analysis.

[CR14] Giraudeau P, Silvestre V, Akoka S (2015). Optimizing water suppression for quantitative NMR-based metabolomics: A tutorial review. Metabolomics.

[CR15] Gronwald W, Klein MS, Kaspar H, Fagerer SR, Nürnberger N, Dettmer K, Bertsch T, Oefner PJ (2008). Urinary metabolite quantification employing 2D NMR spectroscopy. Analytical Chemistry.

[CR16] Häckl M, Tauber P, Schweda F, Zacharias HU, Altenbuchinger M, Oefner PJ, Gronwald W (2021). An R-package for the deconvolution and integration of 1D NMR data: MetaboDecon1D. Metabolites.

[CR17] Jacob D, Deborde C, Lefebvre M, Maucourt M, Moing A (2017). NMRProcFlow: A graphical and interactive tool dedicated to 1D spectra processing for NMR-based metabolomics. Metabolomics.

[CR18] Jacob, D., Deborde, C., & Moing, A. (2020). BioStatFlow-Statistical analysis workflow for" omics" data. Preprint retrieved from arXiv:2007.04599.

[CR19] Jamovi Team. (2021). The jamovi project. jamovi (version 2.2) [computer software]. Retrieved from https://www.jamovi.or.

[CR20] Jung Y-S, Hyeon J-S, Hwang G-S (2016). Software-assisted serum metabolite quantification using NMR. Analytica Chimica Acta.

[CR21] Klein MS, Oefner PJ, Gronwald W (2013). Metaboquant: A tool combining individual peak calibration and outlier detection for accurate metabolite quantification in 1D 1H and 1H-13C HSQC NMR spectra. BioTechniques.

[CR22] Kluyver T, Ragan-Kelley B, Pérez F, Granger BE, Bussonnier M, Frederic J, Kelley K, Hamrick JB, Grout J, Corlay S, Loizides F, Schmidt B (2016). Jupyter Notebooks—A publishing format for reproducible computational workflows. Positioning and power in academic publishing: Players, agents and agendas.

[CR23] Kolokolova, T. N., Savel’ev, O. Y., Sergeev, N. M., Shpigun, O. A., Sokolov, K. V., & Skvortsova, V. I. (2010). Nuclear magnetic resonance spectroscopy in solving the analytical problems of medicine: Analysis of cerebrospinal fluid. *Journal of Analytical Chemistry, *65(10), 1073–1081. 10.1134/S106193481010014X.

[CR24] Krattenmacher F, Heermann T, Calvet A, Krawczyk B, Noll T (2019). Effect of manufacturing temperature and storage duration on stability of chemically defined media measured with LC-MS/MS. Journal of Chemical Technology & Biotechnology.

[CR25] Letertre, M. P., Giraudeau, P., & De Tullio, P. (2021). Nuclear magnetic resonance spectroscopy in clinical metabolomics and personalized medicine: Current challenges and perspectives. *Frontiers in Molecular Biosciences, *8. 10.3389/fmolb.2021.698337. 10.3389/fmolb.2021.698337 PMC8488110 34616770

[CR26] Li, D. W., Bruschweiler-Li, L., Hansen, A. L., & Brüschweiler, R. (2023). DEEP Picker1D and Voigt Fitter1D: A versatile tool set for the automated quantitative spectral deconvolution of complex 1D NMR spectra. *Magnetic Resonance *4, 19–26. 10.5194/mr-4-19-2023. 10.5194/mr-4-19-2023 PMC10539790 37904796

[CR27] Mckay RT (2011). How the 1D-NOESY suppresses solvent signal in metabonomics NMR spectroscopy: An examination of the pulse sequence components and evolution. Concepts in Magnetic Resonance Part A.

[CR28] Melis R, Braca A, Sanna R, Spada S, Mulas G, Fadda ML, Sassu MM, Serra G, Anedda R (2019). Metabolic response of yellow mealworm larvae to two alternative rearing substrates. Metabolomics.

[CR29] Mendez KM, Pritchard L, Reinke SN, Broadhurst DI (2019). Toward collaborative open data science in metabolomics using Jupyter Notebooks and cloud computing. Metabolomics.

[CR30] Monakhova YB, Schäfer H, Humpfer E, Spraul M, Kuballa T, Lachenmeier DW (2011). Application of automated eightfold suppression of water and ethanol signals in 1H NMR to provide sensitivity for analyzing alcoholic beverages. Magnetic Resonance in Chemistry.

[CR31] Nagana Gowda GA, Hong NN, Raftery D (2021). Evaluation of fumaric acid and maleic acid as internal standards for NMR analysis of protein precipitated plasma, serum, and whole blood. Analytical Chemistry.

[CR32] Saude EJ, Sykes BD (2007). Urine stability for metabolomic studies: Effects of preparation and storage. Metabolomics.

[CR33] Schmid, N., Bruderer, S., Paruzzo, F., Fischetti, G., Toscano, G., Graf, D., Fey, M., Henrici, A., Ziebart, V., Heitmann, B., Grabner, H., Wegner, J. D., Sigel, R. K. O., & Wilhelm, D. (2023). Deconvolution of 1D NMR spectra: A deep learning-based approach. *Journal of Magnetic Resonance, *347, 107357. 10.1016/j.jmr.2022.107357. 10.1016/j.jmr.2022.107357 36563418

[CR34] Shimizu A, Ikeguchi M, Sugai S (1994). Appropriateness of DSS and TSP as internal references for 1H NMR studies of molten globule proteins in aqueous media. Journal of Biomolecular NMR.

[CR35] Snytnikova OA, Khlichkina AA, Sagdeev RZ, Tsentalovich YP (2019). Evaluation of sample preparation protocols for quantitative NMR-based metabolomics. Metabolomics.

[CR36] Spierto FW, Hannon WH, Gunter EW, Smith SJ (1997). Stability of urine creatinine. Clinica Chimica Acta.

[CR37] Standage SW, Xu S, Brown L, Ma Q, Koterba A, Lahni P, Devarajan P, Kennedy MA (2021). NMR-based serum and urine metabolomic profile reveals suppression of mitochondrial pathways in experimental sepsis-associated acute kidney injury. American Journal of Physiology-Renal Physiology.

[CR38] Vignoli A, Ghini V, Meoni G, Licari C, Takis PG, Tenori L, Turano P, Luchinat C (2019). High-throughput metabolomics by 1D NMR. Angewandte Chemie International Edition.

[CR39] Wang B, Goodpaster AM, Kennedy MA (2013). Coefficient of variation, signal-to-noise ratio, and effects of normalization in validation of biomarkers from NMR-based metabonomics studies. Chemometrics and Intelligent Laboratory Systems.

[CR40] Wishart DS (2008). Quantitative metabolomics using NMR. TrAC Trends in Analytical Chemistry.

[CR41] Wishart DS, Bigam CG, Yao J, Abildgaard F, Dyson HJ, Oldfield E, Markley JL, Sykes BD (1995). ^1^H, ^13^C and ^15^N chemical shift referencing in biomolecular NMR. Journal of Biomolecular NMR.

[CR42] Wishart DS, Cheng LL, Copié V, Edison AS, Eghbalnia HR, Hoch JC, Gouveia GJ, Pathmasiri W, Powers R, Schock TB (2022). NMR and metabolomics—A roadmap for the future. Metabolites.

[CR43] Wyss M, Kaddurah-Daouk R (2000). Creatine and creatinine metabolism. Physiological Reviews.

[CR44] Zecchini M, Lucas R, Le Gresley A (2019). New insights into the cystine-sulfite reaction. Molecules.

[CR45] Zheng C, Zhang S, Ragg S, Raftery D, Vitek O (2011). Identification and quantification of metabolites in 1H NMR spectra by Bayesian model selection. Bioinformatics.

