# Supplementary material for: NMR metabolite quantification of a synthetic urine sample: an inter-laboratory comparison of processing workflows
Source: Metabolomics. 2023 Jul 7;19(7):65. doi: 10.1007/s11306-023-02028-4 (PMC10328857; doi:10.1007/s11306-023-02028-4)
Supplement: Supplementary file 1 — Supplementary file1 (PDF 574 KB) [file 11306_2023_2028_MOESM1_ESM.pdf]

SUPPLEMENTARY INFORMATION

Metabolomics

Article title: **NMR metabolite quantification of a synthetic urine sample: an inter-laboratory comparison of processing workflows**

Cécile Canlet[1*], Catherine Deborde[2*], Edern Cahoreau[3], Grégory Da Costa[4], Roselyne Gautier[1], Daniel Jacob[2], Cyril Jousse[5], Mélia Lacaze[1], Inès Le Mao[4], Estelle Martineau[6,7], Lindsay Peyriga[3], Tristan Richard[4], Virginie Silvestre[6], Mounir Traïkia[5], Annick Moing[2,$], Patrick Giraudeau[6,$]

[1] Toxalim (Research Centre in Food Toxicology), Toulouse University, INRAE UMR 1331, ENVT, INP-Purpan, UPS, Toulouse, France, Metatoul-AXIOM Platform, National Infrastructure for Metabolomics and Fluxomics: MetaboHUB, Toxalim, INRAE, F-31027, Toulouse, France

[2] INRAE, Univ. Bordeaux, Biologie du Fruit et Pathologie, UMR1332, Metabolome Bordeaux - MetaboHUB, Centre INRAE de Nouvelle-Aquitaine Bordeaux, F-33140 Villenave d'Ornon, France

[3] TBI, Université de Toulouse, CNRS, INRAE, INSA, MetaboHUB-MetaToul, National Infrastructure of Metabolomics & Fluxomics (ANR-11-INBS-0010), F-31077, Toulouse, France

[4] Univ. Bordeaux, Bordeaux INP, INRAE, OENO, UMR 1366, ISVV, Metabolome Bordeaux - MetaboHUB, F-33140 Villenave d'Ornon, France

[5] Université Clermont Auvergne, Clermont Auvergne INP, CNRS, Institut de Chimie de Clermont-Ferrand, F-63000 Clermont–Ferrand, France. Université Clermont Auvergne, INRAE, UNH, Plateforme d'Exploration du Métabolisme, MetaboHUB Clermont, Clermont-Ferrand, France.

[6] Nantes Université, CNRS, CEISAM UMR 6230, F-44000 Nantes, France

[7] CAPACITES SAS, F-44200 Nantes, France

Corresponding authors: annick.moing@inrae.fr, patrick.giraudeau@univ-nantes.fr

Online Resource 1 Preparation and metabolite concentrations of synthetic urine and calibration-range solutions.

Preparation of the solutions

The synthetic urine sample was prepared in ultrapure water by gravimetric method (Sartorius analytical balance, Gottingen, Germany) with known amounts of 32 commercial metabolites. The blank solution was prepared from a potassium phosphate solution (20 mM) supplemented with KCl (100 mM), NaCl (290 mM), NaOH (32 mM), $NaHCO_3$ (11 mM), H_2SO_4 (10 mM) and urea (820 mM) in ultrapure water. In a 50 ml volumetric flask, 25 ml of blank solution were added with volumes of the pure metabolite solutions, and the volume was complemented with ultrapure water. Fifteen ml of synthetic urine were then mixed with 6 ml of sodium and potassium phosphate buffer solution (200

mM, pH 7.4). The pH of 900 µl of the final synthetic urine was adjusted to pH 7.4 ± 0.02 with NaOD or DCl 1-M solutions (1.2 µl added on average) using a titration pH unit (BTpH, Bruker, Karlsruhe, Germany). A volume of 630 µl of this pH-adjusted synthetic urine was supplemented with 70 µl D_2O containing 3-trimethylsilyl-propionic-2,2,3,3-*d4* acid sodium salt (TSP, 258 µM in NMR tube). Depending on the metabolites, the metabolite concentration in the NMR tube ranged from 64 to 7,959 µM. Five calibration-range solutions containing the 32 compounds with concentrations ranging from 0.1 to 12 mM were also prepared and their pH was adjusted. The calibration solutions were also supplemented with TSP for chemical shift calibration.

Metabolite concentrations. Concentrations in the NMR tube (µM).

Metabolites	Synthetic urine (µM)	Cal-1	Cal-2	Cal-3	Cal-4	Cal-5
1-methyl-L-histidine (HMDB0000001 [a] ; CHEBI:70958 [b])	127.66	291.60	207.36	129.60	97.20	64.80
(S)-2-hydroxybutyric acid (HMDB00008 ; CHEBI:1148)	398.06	583.95	486.62	389.30	291.97	194.65
3-methyl-L-histidine (HMDB00479 ; CHEBI:70959)	120.82	206.74	161.52	122.75	96.91	64.61
Acetic acid (HMDB00042 ; CHEBI:15366)	359.13	64.02	192.05	358.49	576.14	768.19
Alanine (HMDB0000042 ; CHEBI:16449)	176.17	259.07	226.69	174.87	129.54	64.77
Allantoin (HMDB0000462 ; CHEBI:15676)	159.68	65.44	111.25	157.06	196.33	242.14
Citric acid (HMDB0000094; CHEBI:30769)	3442.75	4496.13	3853.82	3211.52	1926.91	642.30
Creatine (HMDB0000064 ; CHEBI:16919)	365.70	1028.34	706.99	372.77	224.95	64.27

Creatinine (HMDB0000562 ; CHEBI:16737)	7959.38	3857.53	5786.30	7715.06	10286.75	12858.44
L-Cysteine (HMDB0000574 ; CHEBI:17561)	265.58	128.61	192.92	263.65	321.53	385.83
Dimethylamine (HMDB0000087 ; CHEBI:17170)	323.48	398.54	365.33	318.83	265.69	199.27
Ethanolamine (HMDB0000149 ; CHEBI:16000)	185.76	70.10	126.17	182.25	266.37	350.49
Formic acid (HMDB0000142 ; CHEBI:30751)	163.91	260.18	208.14	162.61	110.58	65.04
Fructose (CHEBI:28757)	63.93	32.29	48.43	64.58	96.86	129.15
D-(+) Glucose (HMDB0000122 ; CHEBI:17634)	247.75	450.45	321.75	244.53	160.88	64.35
L-Glutamine (HMDB0000641 ; CHEBI:18050)	265.34	385.48	321.23	263.41	192.74	128.49
Glycerol (HMDB0000131 ; CHEBI:17754)	160.15	64.58	109.78	161.44	245.39	322.88
Glycine (HMDB0000123 ; CHEBI:15428)	1205.27	3222.64	2255.85	1289.06	966.79	644.53
Guanidoacetic acid (HMDB0000128 ; CHEBI:16344)	706.51	64.23	449.60	770.74	867.09	963.43
Hippuric acid (HMDB0000714 ; CHEBI:18089)	2043.63	4498.56	3213.26	1927.95	1285.30	642.65
L-Histidine (HMDB0000177 ; CHEBI:15971)	482.38	256.59	384.88	481.10	545.24	641.46
Indoxylsulfate (HMDB0000682 ; CHEBI:43355)	156.07	191.89	172.70	153.51	108.74	63.96

Isocitric acid (HMDB0000193 ; CHEBI:30887)	462.96	644.79	548.07	483.59	386.87	257.91
L-Lactic acid (HMDB0000190; CHEBI:422)	96.54	146.08	120.68	95.27	82.57	63.51
L-Lysine (HMDB0000182 ; CHEBI:18019)	144.31	64.71	103.54	142.37	187.67	232.97
Myo-inositol (HMDB0000211 ; CHEBI:17268)	148.81	172.44	159.67	146.90	102.19	63.87
Phenylacetyl-L-glutamine (HMDB0006344; CHEBI:17884)	373.39	64.16	224.55	372.11	481.18	641.57
L-Pyroglutamic acid (HMDB0000267 ; CHEBI:18183)	229.27	449.55	321.11	231.20	160.55	64.22
L-Serine (HMDB0000187 ; CHEBI:17115)	208.79	129.69	162.11	207.50	259.37	324.21
L-Threonine (HMDB0000167 ; CHEBI:16857)	119.51	64.95	90.93	116.91	155.88	194.85
Trigonelline (HMDB0000875 ; CHEBI:18123)	129.72	65.19	162.96	247.71	521.49	880.01
Trimethylamine N-oxide (HMDB0000925 ; CHEBI:15724)	944.69	321.32	642.64	963.96	1285.29	1606.61
Creatine + Creatinine	8325.08	4885.88	6493.28	8087.84	10511.70	12922.71
L-Lactic acid + L -Threonine	216.05	211.03	211.61	212.18	238.45	258.36

[a] HMDB: Human Metabolome Database, identifier from www.hmdb.ca
[b] CHEBI: Chemical Entities of Biological Interest, identifier from https://www.ebi.ac.uk/chebi/init.do

Online Resource 2. Strategies used for processing 1D-zgpr and NOESYpr spectra of urine for compound quantification.

**Strategy code**	**Integration module and mode**	**Calibration mode**	**Number of operators for**	
			**zgpr**	**NOESYpr**
PI1-TSPref	TopSpin, peak integration	internal referencing with TSP	6	
PI1-ExtCal	TopSpin, peak integration	external calibration with calibration-range solutions	6	5
PI2-ExtCal	NMRProcFlow, peak integration	external calibration with calibration-range solutions	1	
DC1-ExtCal	Mnova, deconvolution	external calibration with calibration-range solutions	1	
DC2-ExtCal	NMRDeconvR, deconvolution	external calibration with calibration-range solutions	1	1

Online Resource 3 Spectral regions (left and right limits in ppm) selected for signal integration in synthetic urine and calibration range spectra for metabolite quantification using zgpr spectra with peak integration and external calibration.

	Integration mode and operator													
Metabolite	PI1<br>Op. 1		PI1<br>Op. 2		PI1<br>Op. 3		PI1<br>Op. 4		PI1<br>Op.5		PI1<br>Op.6		PI2<br>Op. 2	
1-methylhistidine	7.010	6.990	7.010	6.992	7.013	6.984	7.013	6.986	7.011	6.986	7.009	6.990	7.010	6.993
2-hydroxybutyric acid	0.930	0.880	1.699	1.616	0.935	0.868	0.937	0.871	0.937	0.866	0.942	0.859	0.925	0.883
3-methylhistidine	7.030	7.010	7.030	7.007	7.039	7.013	7.046	7.013	7.034	7.011	7.032	7.009	7.030	7.012
Acetic acid	1.930	1.915	1.929	1.916	1.93	1.914	1.931	1.917	1.929	1.919	1.929	1.918	1.929	1.918
Alanine	1.500	1.470	1.498	1.47	1.508	1.453	1.504	1.468	1.498	1.468	1.498	1.469	1.498	1.470
Allantoin	5.405	5.390	5.411	5.386	5.412	5.383	5.405	5.387	5.415	5.380	5.406	5.380	5.404	5.390
Citric acid	2.690	2.635	2.691	2.638	2.692	2.635	2.695	2.637	2.695	2.632	2.690	2.634	2.689	2.664
Creatine	3.946	3.925	3.942	3.927	3.943	3.927	3.943	3.928	3.951	3.923	3.944	3.927	3.050	3.032
Creatinine	4.070	4.050	4.067	4.053	4.069	4.047	4.068	4.049	4.070	4.049	4.069	4.052	3.058	3.038
Cystine [a]	ND		ND		ND		ND		ND		ND		3.381	3.366
Dimethylamine	2.735	2.715	2.736	2.717	2.745	2.709	2.739	2.708	2.738	2.716	2.739	2.712	2.738	2.716
Ethanolamine	ND		3.824	3.819	ND		ND		ND		ND		ND	
Formic acid	8.470	8.450	8.478	8.445	8.469	8.453	8.470	8.447	8.489	8.438	8.480	8.440	8.466	8.456
Fructose	3.920	3.890	ND		3.921	3.888	3.922	3.887	ND		3.920	3.889	ND	
Glucose	5.250	5.230	5.249	5.234	5.26	5.226	5.257	5.224	5.253	5.226	5.250	5.226	3.503	3.495
Glutamine	ND		2.471	2.458	ND		ND		ND		ND		2.166	2.158
Glycerol	ND		3.670	3.664	ND		ND		ND		ND		3.656	3.651
Glycine	3.572	3.558	3.570	3.562	3.576	3.559	3.58	3.558	3.570	3.558	3.578	3.558	3.573	3.560
Guanidoacetic acid	3.805	3.795	3.805	3.797	3.804	3.793	3.804	3.798	3.808	3.797	3.807	3.797	3.805	3.795
Hippuric acid	7.670	7.620	7.587	7.528	7.59	7.53	7.582	7.531	7.673	7.604	7.670	7.610	7.662	7.649
Histidine	7.090	7.060	7.090	7.055	7.093	7.062	7.092	7.058	7.092	7.058	7.100	7.050	7.081	7.066
Indoxylsulfate	7.230	7.109	7.524	7.498	7.237	7.181	7.234	7.181	7.236	7.185	7.227	7.180	7.721	7.711
Isocitric acid	3.000	2.951	2.999	2.951	3.002	2.949	3.000	2.952	2.998	2.954	3.004	2.947	2.435	2.427
Lactic acid	ND		4.143	4.132	ND		ND		ND		ND		4.140	4.133
Lysine	ND		3.025	3.017	ND		ND		1.784	1.702	ND		1.887	1.882

Myo-inositol	3.557	3.527	3.542	3.528	3.558	3.526	3.558	3.523			3.558	3.528	3.307	3.301
Phenylacetylglutamine	7.390	7.34	7.451	7.404	7.456	7.398	7.461	7.393	7.462	7.394	7.460	7.395	7.435	7.422
Pyroglutamic acid	2.074	2.002	2.074	2.002	2.485	2.378	2.426	2.383	2.072	2.004	2.073	2.000	2.397	2.392
Serine	ND		3.852	3.844	ND		ND		ND		ND		3.848	3.844
Threonine	4.291	4.23	4.288	4.236	4.285	4.239	4.287	4.234	4.290	4.231	4.286	4.237		
Trigonelline	8.870	8.82	8.866	8.819	8.871	8.808	9.141	9.109	9.156	9.102	9.160	9.100	8.861	8.823
Trimethylamine-N-oxide	3.280	3.26	3.277	3.261	3.28	3.259	3.278	3.26	3.279	3.259	3.277	3.259	3.279	3.259
Lactic acid + Threonine	1.350	1.319	1.347	1.318	1.362	1.307	1.356	1.312	1.357	1.311	1.351	1.305	1.350	1.316

Op., operator; ND, not determined due to lack of quantifiable resonance

[a] Issued from cysteine

Online Resource 4 Spectral regions (left and right limits in ppm) selected for signal integration in synthetic urine and calibration range spectra for metabolite quantification using NOESYpr spectra with peak integration and external calibration.

	Integration mode and operator									
Metabolite	PI1 Op. 1		PI1 Op. 2		PI1 Op. 3		PI1 Op. 4		PI1 Op.6	
1-methylhistidine	7.010	6.990	7.01	6.992	7.013	6.984	7.013	6.986	7.009	6.990
2-hydroxybutyric acid	0.930	0.880	1.699	1.616	0.935	0.868	0.937	0.871	0.942	0.859
3-methylhistidine	7.030	7.010	7.029	7.012	7.039	7.013	7.046	7.013	7.032	7.009
Acetic acid	1.930	1.915	1.929	1.916	1.930	1.914	1.931	1.917	1.929	1.918
Alanine	1.500	1.470	1.498	1.47	1.508	1.453	1.504	1.468	1.498	1.469
Allantoin	5.405	5.390	5.411	5.386	5.413	5.383	5.405	5.387	5.406	5.380
Citric acid	2.690	2.635	2.691	2.638	2.692	2.635	2.695	2.637	2.690	2.634
Creatine	3.946	3.925	3.942	3.927	3.943	3.927	3.943	3.928	3.944	3.927
Creatinine	4.070	4.500	4.067	4.053	4.070	4.047	4.068	4.049	4.069	4.052
Cystine [a]	ND		ND		ND		ND		ND	
Dimethylamine	2.735	2.715	2.736	2.717	2.745	2.709	2.739	2.708	2.739	2.712
Ethanolamine	ND		3.824	3.819	ND		ND		ND	
Formic acid	8.470	8.450	8.478	8.445	8.469	8.453	8.470	8.447	8.480	8.440
Fructose	3.920	3.890			3.921	3.888				
Glucose	5.250	5.230	5.249	5.234	5.251	5.228	5.257	5.224	5.250	5.226
Glutamine	ND		2.471	2.458	ND		ND		ND	
Glycerol	ND		3.67	3.664	ND		ND		ND	
Glycine	3.572	3.558	3.57	3.562	3.576	3.559	3.570	3.558	3.578	3.558
Guanidoacetic acid	3.805	3.795	3.805	3.797	3.804	3.793	3.804	3.798	3.807	3.797
Hippuric acid	7.670	7.620	7.587	7.528	7.590	7.530	7.582	7.531	7.670	7.610
Histidine	7.090	7.060	7.090	7.055	7.094	7.062	7.092	7.058	7.100	7.050
Indoxylsulfate	7.230	7.190	7.524	7.498	7.238	7.181	7.226	7.190	7.310	7.260
Isocitric acid	3.000	2.951	2.999	2.951	3.002	2.949	3.000	2.952	3.004	2.947
Lactic acid	ND		4.143	4.132	4.143	4.110	ND		1.351	1.305
Lysine	ND		3.025	3.017	ND		ND		ND	

Myo-inositol	3.557	3.527	3.542	3.528	3.557	3.526	3.558	3.523	3.558	3.528
Phenylacetylglutamine	7.390	7.340	7.451	7.404	7.456	7.398	7.450	7.400	7.460	7.395
Pyroglutamic acid	2.074	2.002	2.074	2.002	2.485	2.378	2.075	2.004	2.073	2.000
Serine	ND		3.852	3.844	ND		ND		ND	
Threonine	4.291	4.230	4.288	4.236	4.285	4.239	4.287	4.234	4.286	4.237
Trigonelline	8.870	8.820	8.866	8.819	8.872	8.808	9.141	9.109	9.160	9.100
Trimethylamine-N-oxide	3.280	3.260	3.277	3.261	3.279	3.259	3.278	3.260	3.277	3.259
Lactic acid + Threonine	1.350	1.319	1.347	1.318	1.362	1.307	1.356	1.312	1.351	1.305

Op., operator; ND, not determined due to lack of quantifiable resonance

[a] Issued from cysteine

Online Resource 5 Comparison of zgpr and NOESYpr spectra of synthetic urine. Zoom in on the 2.7-1.4 and 7.78-6.92 ppm regions of representative spectra. red, zgpr spectrum. blue, NOESYpr spectrum; green difference between the two spectra.

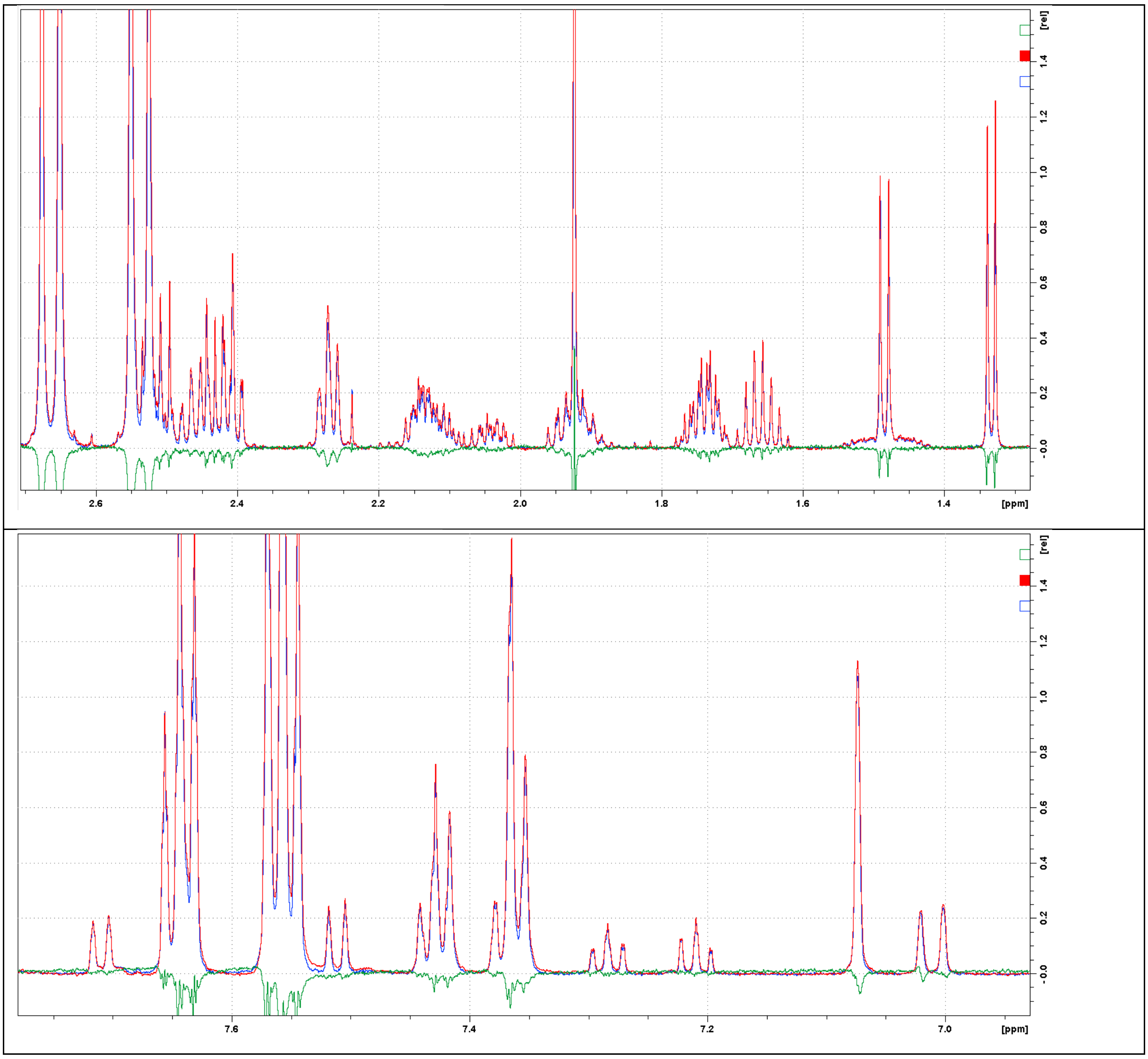

Online Resource 6 Representative 1D ^{1}H-NMR noesypr1d spectrum of synthetic urine with metabolite annotation. Numbers indicate the following metabolites: 1: 1-methylhistidine; 2: 2-hydroxybutyric acid; 3: 3-methylhistidine; 4: acetic acid; 5: alanine; 6: allantoin; 7: citric acid; 8: creatine; 9: creatinine; 10: dimethylamine; 11: ethanolamine; 12: formic acid; 13: fructose; 14: glucose; 15: glutamine; 16: glycerol; 17: glycine; 18: guanidoacetic acid; 19: hippuric acid; 20: histidine; 21: indoxylsulfate; 22: isocitric acid; 23: lactic acid; 24: lysine; 25: myo-inositol; 26: phenylacetylglutamine; 27: pyroglutamic acid; 28: serine; 29: threonine; 30: trigonelline; 31: trimethylamine-N-oxide.

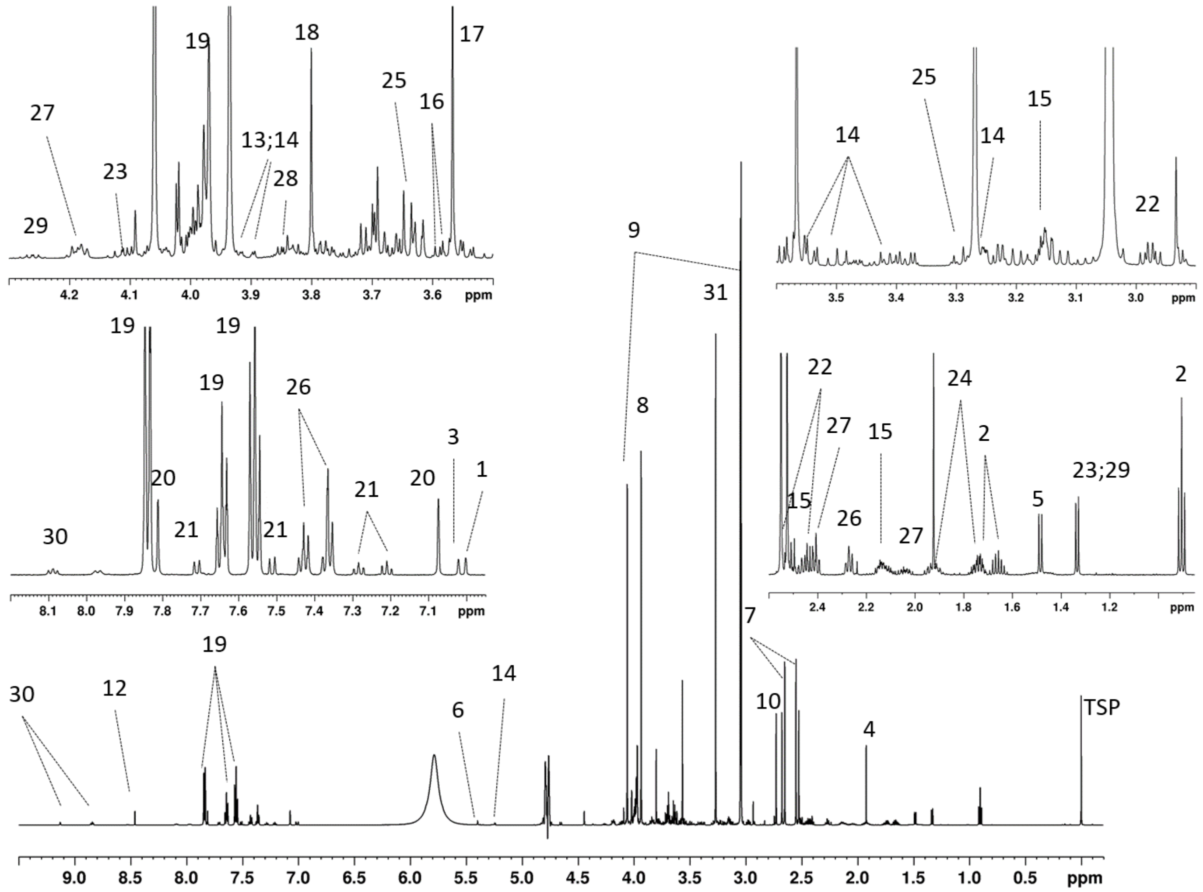

Online Resource 7 Metabolites quantified from synthetic-urine zgpr spectra using different strategies for resonance integration and calibration. Pink, peak integration with Topspin and internal calibration (PI1-TSPref); blue, peak integration with Topspin and external calibration (PI1-ExtCal); green, peak integration with NMRProcFlow and external calibration (PI2-ExtCal); orange, resonance deconvolution with Mnova and external calibration (DC1-ExtCal); red, resonance deconvolution with NMRDeconvR and external calibration (DC2-ExtCal).

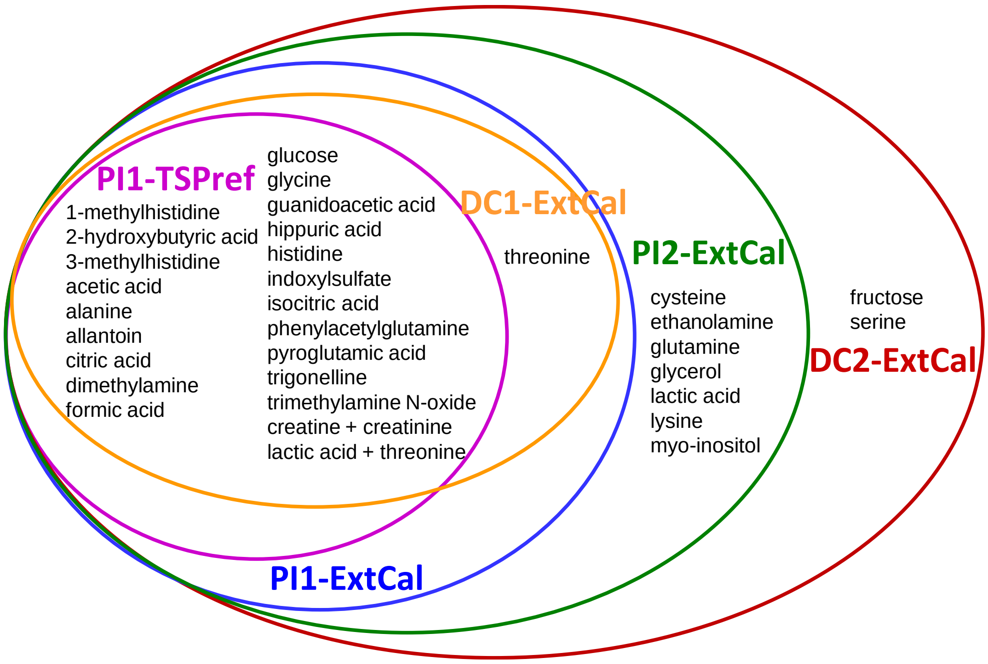

Online Resource 8 PCA of quantification data of 20 metabolites and two sums of two metabolites of synthetic urine quantified in zgpr spectra by all spectra processing strategies. (A) Scores plot. Squares, TopSpin and TSP referencing (PI1-TSPref); circles, two methods of integration (PI1, TopSpin; PI2, NMRProcFlow) or deconvolution (DC1, Mnova; DC2, NMRDeconvR) with external calibration; triangle, plot of the theoretical sample (Theor). (B) Loadings plot with metabolite annotation.

A

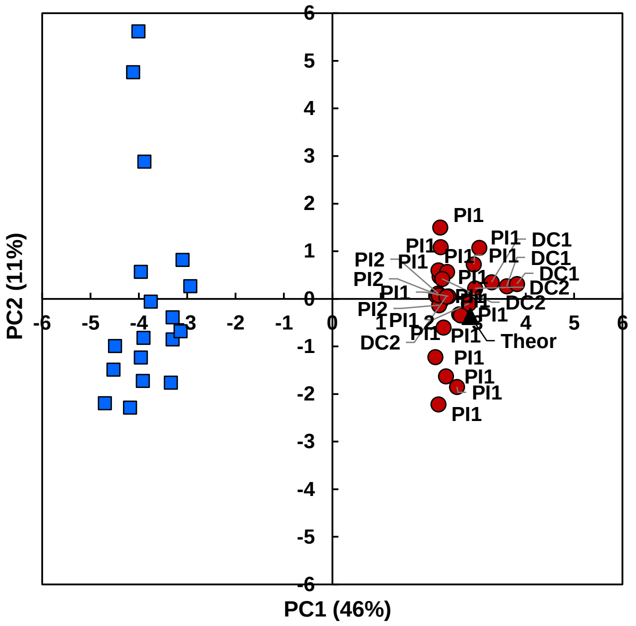

B

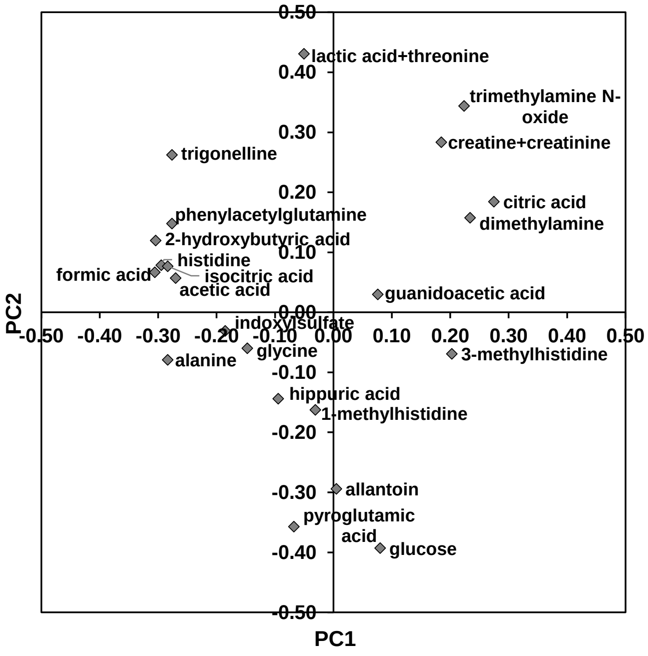

Online Resource 9 Kruskal-Wallis test for operator effect for quantification of 20 metabolites and two sums of metabolites using zgpr spectra and TopSpin integration with external calibration.

Metabolites	χ^2	df	p-value
1-methylhistidine	7.924	5	1.600E-01
2-hydroxybutyric acid	0.111	5	1.000E+00
3-methylhistidine	12.766	5	2.600E-02
Acetic acid	3.770	5	5.830E-01
Alanine	2.637	5	7.560E-01
Allantoin	0.813	5	9.760E-01
Citric acid	3.129	5	6.800E-01
Dimethylamine	7.924	5	1.600E-01
Formic acid	4.462	5	4.850E-01
Glucose	0.602	5	9.880E-01
Glycine	15.971	5	7.000E-03
Guanidoacetic acid	12.743	5	2.600E-02
Hippuric acid	12.813	5	2.500E-02
Histidine	8.020	5	1.550E-01
Indoxylsulfate	2.591	5	7.630E-01
Isocitric acid	3.339	5	6.480E-01
Phenylacetylglutamine	0.298	5	9.980E-01
Pyroglutamic acid	11.901	5	3.600E-02
Trigonelline	0.953	5	9.660E-01
Trimethylamine N-oxide	9.515	5	9.000E-02
Creatine+Creatinine	1.795	5	8.770E-01
Lactic acid+Threonine	14.778	5	1.100E-02

df, degrees of freedom

Online Resource 10 Precision for 21 or 29 metabolites and two sums of metabolites quantified from synthetic-urine NOESYpr spectra using two spectra processing strategies, peak integration with TopSpin (PI1) or peak deconvolution with NMRDeconvR (DC2) performed by five or one operators (op.), and external calibration (ExtCal).

	Precision (CV %)		
Metabolite	PI1-ExtCal		DC2-ExtCal
	Mean value (n=5 op.)	SD	Value (n=1 op.)
1-methylhistidine	2.19	1.29	5.06
2-hydroxybutyric acid	0.20	0.05	0.71
3-methylhistidine	1.31	0.24	2.57
Acetic acid	0.22	0.01	3.53
Alanine	0.23	0.05	1.85
Allantoin	26.99	4.37	3.01
Citric acid	0.14	0.005	0.92
Cysteine [a]	ND		9.91
Dimethylamine	0.36	0.03	1.82
Ethanolamine	ND		0.96
Formic acid	1.16	0.10	2.33
Glucose	42.60	12.11	4.81
Glutamine	ND		3.12
Glycerol	ND		4.42
Glycine	0.26	0.10	1.94
Guanidoacetic acid	0.52	0.03	2.93
Hippuric acid	0.21	0.12	0.66
Histidine	0.67	0.16	2.53
Indoxylsulfate	2.56	0.16	3.35
Isocitric acid	1.64	0.05	2.56
Lactic acid	ND		4.20
Lysine	ND		4.20
Myo-inositol	1.71	0.19	3.26
Phenylacetylglutamine	0.50	0.08	1.72
Pyroglutamic acid	2.83	1.30	4.05
Serine	ND		4.28
Threonine	ND		6.79
Trigonelline	1.42	0.35	3.82
Trimethylamine-N-oxide	0.24	0.00	2.04
Creatine + Creatinine	3.01	0.01	3.81
Lactic acid + Threonine	1.05	1.68	1.93

op., operator; SD, standard deviation; ND, not determined due to lack of quantifiable resonance
[a] Estimated from cystine

Online Resource 11 Relative trueness for 21 or 29 metabolites and two sums of metabolites quantified from synthetic-urine NOESYpr spectra using two spectra processing strategies, peak integration with TopSpin (PI1) or peak deconvolution with NMRDeconvR (DC2) performed by five or one operators, and external calibration.

	Relative trueness (%)		
Metabolite	PI1-ExtCal Mean value (n=5 op.)	SD	DC2-ExtCal Value (n=1 op.)
1-methylhistidine	-4.28	7.87	3.41
2-hydroxybutyric acid	3.35	0.63	5.21
3-methylhistidine	3.81	3.95	3.39
Acetic acid	2.50	0.05	4.86
Alanine	0.81	0.13	1.45
Allantoin	5.99	0.09	6.77
Citric acid	1.40	0.01	-3.14
Cysteine [a]	ND		-3.65
Dimethylamine	0.95	1.37	4.09
Ethanolamine	ND		3.47
Formic acid	-3.23	0.12	0.78
Glucose	9.93	7.16	-6.38
Glutamine	ND		-4.95
Glycerol	ND		-3.26
Glycine	-0.71	1.49	5.08
Guanidoacetic acid	-1.25	0.41	-1.63
Hippuric acid	0.72	0.40	0.42
Histidine	3.11	0.19	2.04
Indoxylsulfate	-0.08	1.87	1.09
Isocitric acid	-5.69	0.44	-0.31
Lactic acid	ND		8.04
Lysine	2.94		2.67
Myo-inositol	-18.47	0.68	-0.88
Phenylacetylglutamine	0.85	0.09	0.37
Pyroglutamic acid	-6.55	5.69	-2.13
Serine	ND		7.83
Threonine	ND		4.30
Trigonelline	-0.43	0.36	1.13
Trimethylamine-N-oxide	1.99	0.32	5.63
Creatine + Creatinine	-4.75	3.85	1.45
Lactic acid + Threonine	8.36	14.53	5.97

op., operator; SD, standard deviation; ND, not determined due to lack of quantifiable resonance
[a] Estimated from cystine
